# The impact of inoculation with the inactivated COVID-19 vaccine on the outcomes of *in vitro* fertilization and embryo transfer: a cohort study of 1,258 women from Sichuan, China

**DOI:** 10.3389/fendo.2025.1491259

**Published:** 2025-06-10

**Authors:** Jia-jing Wei, Yu Qiu, Mei Leng, Fu-rui Chen, Mei-yu Liang, Xi Deng, Rong-ning Ma, Jing Hei, Jesse Li-Ling, Yan Gong

**Affiliations:** ^1^ Reproductive Medicine Centre, Sichuan Provincial Women’s and Children’s Hospital, The Affiliated Women’s and Children’s Hospital of Chengdu Medical College, Chengdu, Sichuan, China; ^2^ School of Clinical Medicine, Chengdu Medical College, Chengdu, Sichuan, China; ^3^ Center of Medical Genetics, West China Second University Hospital, Sichuan University, Chengdu, Sichuan, China

**Keywords:** SARS-CoV-2, inactivated vaccine, *in vitro* fertilization, embryo transfer, live birth

## Abstract

**Objective:**

This study aimed to assess the impact of inoculation with the inactivated coronavirus disease 2019 (COVID-19) vaccine on the outcomes of *in vitro* fertilization and embryo transfer (IVF-ET).

**Methods:**

From January 2021 to December 2022, patients undergoing their first cycle of IVF-ET at the Reproductive Medicine Center of Sichuan Provincial Women’s and Children’s Hospital were prospectively enrolled. Based on inoculation with inactivated COVID-19 vaccines before ovarian stimulation (OS) by a gonadotrophin-releasing hormone (GnRH) antagonist or agonist protocol, the patients were divided into the vaccinated group (*n* = 713) and the unvaccinated group (*n* = 545). The vaccinated group were sub-grouped based on the dose of inoculation (single dose, *n* = 74; double dose, *n* = 275; and triple dose, *n* = 126) and the interval between the first inoculation and OS (<3 months, *n* = 65; 3–6 months, *n* = 123; and >6 months, *n* = 287).

**Results:**

The rates of mature oocytes, normal fertilization, cleavage embryo, high-quality cleavage embryo, blastocysts, and high-quality blastocysts were not significantly different between the vaccinated and unvaccinated groups (*p* > 0.05). For fresh embryo transfer, the implantation rate (IR), the clinical pregnancy rate (CPR), the live birth rate (LBR), the gestational age at delivery, and the birth weight of infants were not significantly different between the two groups (*p* > 0.05). The IR, CPR, LBR, and birth weight of infants were not significantly different for both the dose and interval subgroups (*p* > 0.05).

**Conclusion:**

Inactivated COVID-19 vaccines may not affect the outcomes of IVF-ET.

## 1 Introduction

Since the end of 2019, the coronavirus disease 2019 (COVID-19) pandemic caused by the severe acute respiratory syndrome coronavirus 2 (SARS-CoV-2) virus had become a global issue, which severely burdened the world’s public health and economy. Consequently, various vaccine types have been designed and manufactured (1, 2). The inactivated COVID-19 vaccine is produced from the virus killed by chemical or physical methods to eliminate the risk of viral reversion (3). The attenuated virus vaccine is produced from viruses with decreased pathogenesis and may induce a strong immune response; however, a major concern from it has been the toxicity after vaccination (4). For viral vector vaccines, adenovirus is used to insert the COVID-19 viral gene into the human body. This vaccine is safe and effective, but the construction of the adenovirus vector is challenging (5). For the messenger ribonucleic acid (mRNA) vaccine, the risk of viral infection is low, and it has the virtue for being economical and effective (6).

Compared with non-pregnant women, pregnant women infected with SARS-CoV-2 have shown great risks of mechanical ventilation, intensive care unit admission, and death (7). Worsening maternal infection may in turn result in adverse neonatal outcomes associated with preterm birth (8, 9). Fortunately, vaccination of the mother could provide passive immunization for the fetus through the placenta (10, 11). Therefore, since the end of 2020, the World Health Organization and other international health institutions have granted approval for the inoculation of the COVID-19 vaccine in women who are pregnant, breastfeeding, or planning to conceive naturally or by assisted reproductive technology (ART) (12). Studies found that COVID-19 mRNA vaccination did not affect the outcomes of ART (13–21). The ovarian reserve, the response to ovarian stimulation (OS), and the outcomes of early pregnancy were not affected by the COVID-19 mRNA vaccine during *in vitro* fertilization and embryo transfer (IVF-ET) (13–16). Neither SARS-CoV-2 infection or the mRNA vaccine nor the immune response to these has detrimentally affected the function of follicles, manifested as a measurable change of the heparan sulfate proteoglycans (the major estrogen-binding protein) in the follicular fluid (17–19). The serum level of the anti-Müllerian hormone (AMH), the endometrial receptivity, and the sustained implantation rate (IR) were not affected by the COVID-19 mRNA vaccine (16, 20). Miller et al. (21) reported that the live birth rates (LBRs), the gestational weeks, and the birth weights were similar between those with or without COVID-19 mRNA vaccination (*n* = 38 and 10, respectively).

The inactivated COVID-19 vaccine comprised almost half of all doses vaccinated globally and has been crucial in fighting the COVID-19 pandemic. In China, it was also the most widely used and has been proven to be safe for individuals over 18 years (22). Some have reported that the ovarian response, the embryo quality, and the ongoing pregnancy rates were not affected by the inactivated COVID-19 vaccine during IVF-ET (23–26). There are also other reports that the inactivated COVID-19 vaccine did not undermine the biochemical pregnancy rates, the clinical pregnancy rates (CPRs), and the abortion rates during frozen–thawed embryo transfer (FET) cycles (27, 28). LBR is an important outcome in IVF-ET; however, only one study has reported that it was not affected by the inactivated COVID-19 vaccine in IVF-ET, but without information on infants and subgroup analysis by interval or dose of vaccination (29). From April 2021 to January 2023, there was a widespread mass vaccination campaign in China using the inactivated COVID-19 vaccine. By now, all pregnant women inoculated with the inactivated vaccine have delivered (or terminated their pregnancy). Against this backdrop, we have the opportunity to compare the LBR and the outcomes of IVF-ET treatment and to analyze the subgroups that had different doses and intervals of vaccination.

## 2 Materials and methods

### 2.1 Study populations

Patients aged 20–45 years undergoing their first IVF-ET treatment at the Reproductive Medicine Center of Sichuan Provincial Women’s and Children’s Hospital from January 2021 to December 2022 were prospectively enrolled. The OS protocol was the gonadotrophin-releasing hormone (GnRH) antagonist or agonist protocol. The exclusion criteria were: history of SARS-CoV-2 infection; oocyte and/or sperm donation; pre-implantation genetic testing (PGT); oocyte frozen; adenomyosis, submucosal, or intramural uterine fibroids; uterine abnormalities; and inaccurate information on vaccination. The patients were divided into the vaccinated group (CoronaVac, an inactivated COVID-19 vaccine produced by Sinovac Biotech Ltd., Beijing, China) and the unvaccinated group based on the inoculation status before OS.

**Figure 1:**
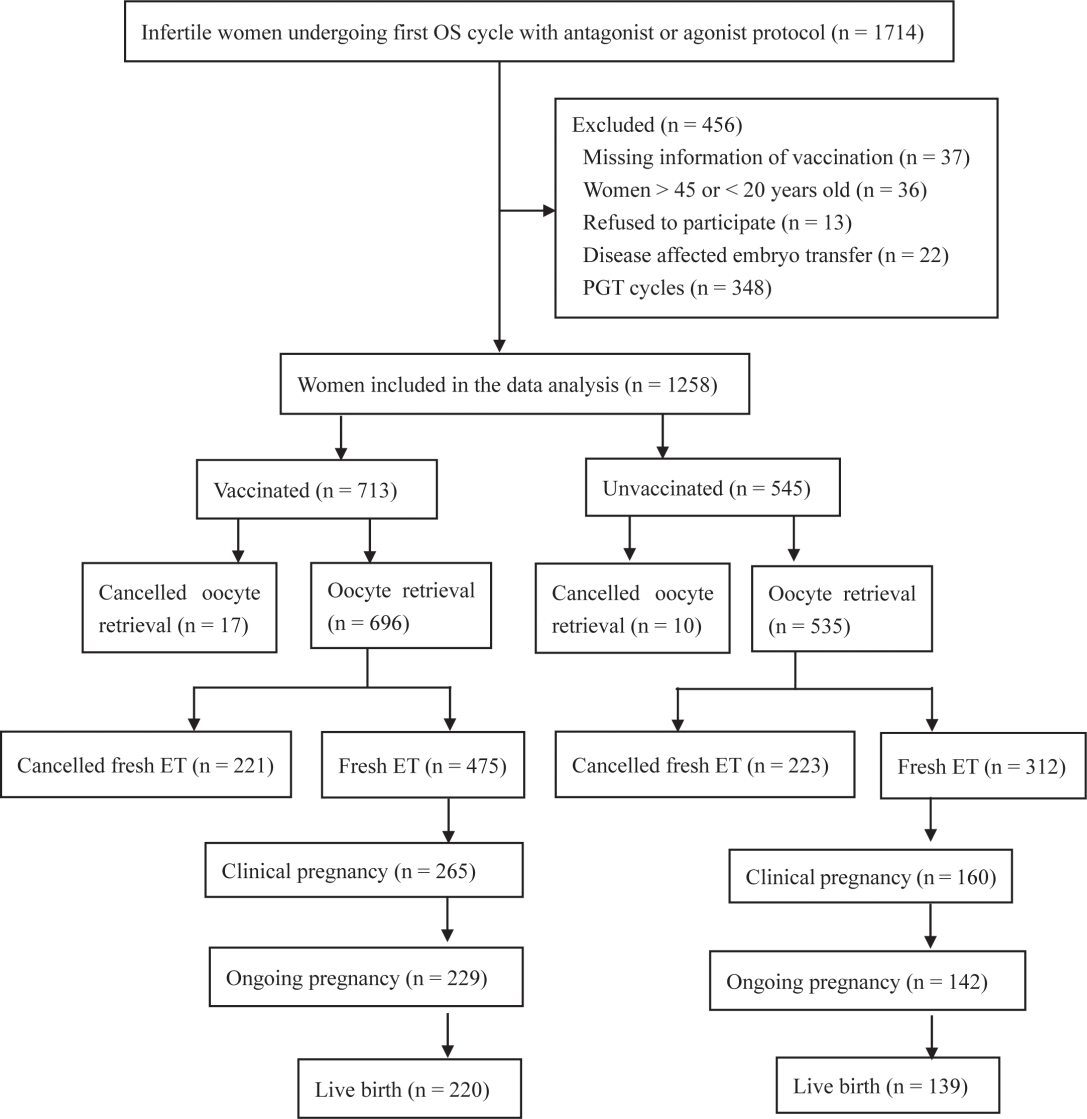
Flow chart for participants recruitment of this study.

The vaccinated group was sub-grouped based on the dose of inoculation (single dose, double dose, and triple dose) or the interval between the first inoculation and OS (<3 months, 3–6 months, and >6 months). In China, the full course of vaccination consists of three doses: the first dose, the second dose (given 1 month after the first dose), and the third dose (a booster dose, given 6 months after the second dose). Prior to the OS, information on the vaccination (i.e., date of vaccination, type, and manufacturer) was recorded by a nurse via Tianfutong (an application program of the Sichuan health database) installed on mobile phones. For all patients, nucleic acid is routinely detected to exclude SARS-CoV-2 infection before OS. The flowchart is shown in **Figure 1**. This prospective cohort study was approved by the Medical Ethics Committee of Sichuan Provincial Women’s and Children’s Hospital. All procedures in this study complied with the ethical standards of the relevant national and institutional committees on human experimentation and the Helsinki Declaration 1975 (2013 revision). The trial was registered in the Chinese Clinical Trial Registry (ChiCTR2200055721; https://www.chictr.org.cn/,16 January 2022).

The body mass index (BMI), the antral follicle count (AFC), and the serum levels of the follicle-stimulating hormone (FSH), luteinizing hormone (LH), estradiol (E_2_), progesterone (P), total testosterone (TT), prolactin, and AMH were measured as described previously (30).

### 2.2 Ovarian stimulation

Patients with decreased ovarian reserve (DOR) or polycystic ovary syndrome (PCOS) were mainly treated with the GnRH antagonist protocol, while others were generally treated with the long-acting GnRH agonist protocol.

With the GnRH antagonist protocol, 125–300 IU/day of recombinant FSH (rFSH) (Gonal-F, Merck-Serono KGaA, Darmstadt, Germany; Jinsai Heng, Jinsai Pharmaceuticals, China; Puregon^®^, Merck Sharp & Dohme, Rahway, NJ, USA) was injected daily from day 2 to day 3 of the menstrual cycle until the trigger day. The rFSH dose was determined based on the AFC, AMH, BMI, and age of the patient and was adjusted according to follicle development and the serum level of E_2_. When the diameter of the leading follicle reached 12 mm or the serum level of LH ≥10 mIU/ml, 0.25 mg GnRH antagonist (Ganirelix, Ocalon, FL, USA) was subcutaneously injected daily until the trigger day. When the diameter of at least one or two follicles has reached 18 mm, 250 μg of recombinant human chorionic gonadotrophin (rHCG; Merck-Sheranova, Darmstadt, Germany) or 0.2 mg of triptorelin (Jinsai Pharmaceuticals, Changchun, China) was injected as the trigger, with the latter used only for all frozen embryo cycles. Transvaginal oocyte retrieval was performed 36.5 h later.

With the GnRH agonist protocol, 3.75 mg of leuprorelin acetate (Beiyi; Shanghai Livzon Pharmaceutical Co., Ltd., Shanghai, China) was injected subcutaneously from day 2 to day 3 of the menstrual cycle. After 28–38 days, 125–300 IU/day rFSH was injected daily when the diameter of most follicles was 5 mm and with serum levels of E_2_ <50 pg/ml and LH and FSH <5 mIU/ml. The standard of the rFSH initiation dose and adjusted dose and the timing of the trigger and ovulation were consistent with the GnRH antagonist protocol.

Oocyte retrieval was cancelled for any of the following conditions: follicular growth failure (10 days after OS, diameter of the leading follicle <10 mm), premature ovulation before oocyte retrieval, and personal reasons. Ovarian hyperstimulation syndrome (OHSS) was diagnosed and graded according to Navot et al. (31).

### 2.3 *In vitro* fertilization and embryo culture

*In vitro* fertilization (IVF) was carried out 39 h after the trigger for all retrieved oocytes, while intracytoplasmic sperm injection (ICSI) was carried out 3–4 h after the retrieval of mature oocytes. A mature oocyte was defined as being at the metaphase II (MII) stage with the first polar body visible in the cytoplasm. A normal fertilized oocyte was confirmed as containing two pronuclei (2PN). The embryo was cultured to cleavage and blastocyst stage in sequential G1-plus/G2-plus medium (Vitrolife, Gothenburg, Sweden) at 37°C in a culture environment containing 6.0% CO_2_ and 5% O_2_. Day 3 cleavage embryo was scored based on the number of blastomeres and the degree of fragmentation, with high-quality embryo categorized as grade I or II (32). On day 5 or day 6, morphological scoring was carried out based on the Gardner and Schoolcraft’s system (32). Blastocysts were considered as usable with a grade over 4CC and as high quality for those over 4BB.

### 2.4 Fresh embryo transfer

Most of the patients were transferred with two cleavage embryos with the highest morphological scores. One embryo was transferred for those with a scarred uterus or with a body height of <1.5 m, and one blastocyst was transferred when eligible (with the number of high-quality cleavage embryos ≥3); otherwise, one cleavage embryo was transferred. Luteal phase support was started on the day after oocyte retrieval by daily injection of 60 mg progesterone oil (Zhejiang Xianju Pharmaceutical Co., Ltd., Taizhou, China) or 90 mg vaginal progesterone (Crinone 8% gel; Merck, Darmstadt, Germany), and 30 mg dydrogesterone (Duphaston; Abbott Healthcare Products B.V., Weesp, the Netherlands) was taken daily.

Embryo implantation, clinical pregnancy, and early miscarriage were defined as previously described (30). Ectopic pregnancy was defined as implantation at any site out of the uterine cavity. Late miscarriage was defined as loss of pregnancy between the 12th and 28th weeks of gestation. Ongoing pregnancy was defined as the detection of fetal heartbeat at the 12th week of gestation. Live birth was defined as delivery of a live fetus after 28 weeks of gestation.

Fresh embryo transfer was cancelled for any of the following conditions: failed oocyte retrieval, no transplantable embryo formation, serum level of P >1.5 ng/ml on the trigger day, diagnosis of OHSS, prevention of OHSS (with number of oocytes retrieved >18 or serum level of E_2_ >5,000 pg/ml on the trigger day), and personal reasons. All of the embryos were frozen, and frozen–thawed embryo was transferred at least 1 month later. Only the outcomes of fresh embryo transfer were analyzed.

### 2.5 Outcomes

The primary outcome was the LBR. The secondary outcomes were the rates of MII, 2PN, cleavage embryo, high-quality cleavage embryo, blastocyst, high-quality blastocyst, implantation, clinical pregnancy, early miscarriage, and ongoing pregnancy.

The above outcomes were calculated as follows: MII rate = number of MII oocytes/number of retrieved oocytes; 2PN rate = number of 2PN oocytes/number of oocytes for fertilization; cleavage embryo rate = number of D3 cleavage embryos/number of cleaved embryos on day 2; high-quality cleavage embryo rate = number of high-quality D3 cleavage embryos/number of cleaved embryos on day 2; blastocyst rate = number of blastocysts/number of D3 cleavage embryos cultured for blastocyst; high-quality blastocyst rate = number of high-quality blastocysts/number of D3 cleavage embryos cultured for blastocyst; IR = number of gestational sacs/number of transferred embryos; CPR = number of clinical pregnancy cycles/number of fresh embryo transfer cycles; ongoing pregnancy rate = number of ongoing pregnancy cycles/number of fresh embryo transfer cycles; early miscarriage rate = number of early miscarriage cycles/number of clinical pregnancy cycles; and LBR = number of live birth cycles/number of fresh embryo transfer cycles.

### 2.6 Statistical analysis

SPSS v26.0 software (IBM, Armonk, NY, USA) was used for statistical analysis. Continuous variables are expressed as the mean ± standard deviation and were analyzed using one-way ANOVA. Categorical variables are expressed as frequency and were compared using chi-square or Fisher’s exact test. Two-tailed *p* < 0.05 was considered as statistically significant, as we had no prior evidence indicating the effect of vaccination on the outcomes of IVF-ET.

## 3 Results

### 3.1 Baseline characteristics of the study population

A flowchart of the recruitment is shown in **Figure 1**. Infertile women undergoing their first OS cycle with an antagonist or agonist protocol (*n* = 1714) were included as participants. Women with missing information on vaccination (*n* = 37), women >45 or <20 years old (*n* = 36), those who refused to participate (*n* = 13), and those with disease-affected embryo transfer (*n* = 22) and PGT cycles (*n* = 348) were excluded. Finally, 1,258 patients were enrolled. They were divided into the vaccinated group (*n* = 713) and the unvaccinated group (*n* = 545) according to their vaccination status before OS.

The age, duration, type and etiology of infertility, BMI, AFC, and serum levels of AMH and basal sex hormones were not significantly different between the vaccinated and unvaccinated groups (*p* > 0.05) (**Table 1**).

**Table 1:** Baseline clinical characteristics of the vaccinated and unvaccinated groups.

Clinical characteristics	Vaccinated group (n = 713)	Unvaccinated group (n = 545)	*P* value
Age (year)	31.85 ± 4.64	31.51 ± 4.43	0.184
Duration of infertility (year)	3.55 ± 2.99	3.31 ± 2.98	0.148
Type of infertility (%)			0.293
Primary	49.23% (351/713)	46.24% (252/545)	
Secondary	50.77% (362/713)	53.76% (293/545)	
Etiology of infertility (%)			0.368
Tubal factor	22.02% (157/713)	16.88% (92/545)	
Ovulatory disorder	4.07% (29/713)	4.04% (22/545)	
Endometriosis	0.84% (6/713)	1.28% (7/545)	
DOR	3.37% (24/713)	3.67% (20/545)	
Male factor	11.36% (81/713)	11.74% (64/545)	
Mixed factor	51.89% (370/713)	54.13% (295/545)	
Unexplained infertility	6.45% (46/713)	8.26% (45/545)	
BMI (kg/m^2^)	22.37 ± 2.87	22.21 ± 2.76	0.304
AFC	15.01 ± 8.47	15.01 ± 8.75	0.989
AMH (ng/mL)	3.31 ± 2.99	3.54 ± 3.04	0.175
Basal FSH (mIU/mL)	6.91 ± 2.64	6.97 ± 2.93	0.713
Basal LH (mIU/mL)	5.28 ± 3.27	5.28 ± 2.94	0.995
Basal E_2_ (pg/mL)	39.29 ± 17.56	38.94 ± 17.36	0.723
Basal P (ng/mL)	0.45 ± 0.18	0.44 ± 0.19	0.234
TT (ng/mL)	0.26 ± 0.16	0.27 ± 0.17	0.641
PRL (uIU/mL)	333.77 ± 127.78	333.04 ± 118.95	0.953

DOR, decreased ovarian reserve; BMI, body mass index; AFC, antral follicle count; AMH, anti-Müllerian hormone; FSH, follicle-stimulating hormone; LH, luteinizing hormone; E_2_, estradiol; P, progesterone; TT, total testosterone; PRL, prolactin.

### 3.2 Outcomes of OS and embryo culture

The proportion of OS protocol; the types of trigger and fertilization; the dose and duration of rFSH; the serum levels of E_2_ and P on the trigger day; the numbers of retrieved oocytes, MII, 2PN, cleavage embryos, and high-quality cleavage embryos; the rates of MII, 2PN, cleavage embryo, high-quality cleavage embryo, blastocyst, and high-quality blastocyst; the incidence of mild, moderate, and severe OHSS; and the rates of cancelled oocyte retrieval were not significantly different between the vaccinated and unvaccinated groups (*p* > 0.05) (**Table 2**).

**Table 2:** Outcomes of OS and embryo culture of the vaccinated and unvaccinated groups.

Variables	Vaccinated (n = 713)	Unvaccinated (n = 545)	*P* value
OS protocol (%)			0.198
GnRH antagonist	43.62% (311/713)	40.00% (218/545)	
GnRH agonist	56.38% (402/713)	60.00% (327/545)	
rFSH duration (day)	10.51 ± 1.82	10.67 ± 1.87	0.130
rFSH dose (IU)	2367.84 ± 795.28	2291.90 ± 895.05	0.114
Serum E_2_ on the trigger day (pg/mL)	2748.82 ± 2165.37	2823.79 ± 2029.01	0.537
Serum P on the trigger day (ng/mL)	1.11 ± 0.67	1.11 ± 0.79	0.952
Type of trigger (%)			0.676
rHCG	93.04% (655/704)	92.42% (500/541)	
triptorelin	6.96% (49/704)	7.58% (41/541)	
Type of fertilization (%)			0.116
IVF	77.28% (534/691)	80.98% (430/531)	
ICSI	22.72% (157/691)	19.02% (101/531)	
Oocytes retrieved	11.55 ± 7.24	11.91 ± 7.27	0.393
MII oocytes	9.61 ± 6.25	10.02 ± 6.22	0.254
2PN oocytes	6.41 ± 4.49	6.56 ± 4.68	0.564
Cleavage embryos	6.39 ± 4.32	6.70 ± 4.74	0.233
High-quality cleavage embryos	1.97 ± 2.22	2.21 ± 2.64	0.087
MII rate (%)	82.56% (6638/8040)	83.49% (5319/6371)	0.142
2PN rate (%)	57.44% (4430/7712)	56.88% (3485/6127)	0.506
Cleavage embryo rate (%)	87.01% (4413/5072)	86.50% (3556/4111)	0.475
High-quality cleavage embryo rate (%)	26.83% (1362/5072)	28.53% (1173/4111)	0.073
Blastocyst rate (%)	50.31% (1603/3186)	51.69% (1329/2571)	0.298
High-quality blastocyst rate (%)	15.47% (493/3186)	14.51% (373/2571)	0.308
Incidence of OHSS (%)			
mild	2.52% (18/713)	3.49% (19/545)	0.317
moderate	1.82% (13/713)	0.73% (4/545)	0.097
severe	0.70% (5/713)	0.92% (5/545)	0.669
Cancelled oocyte retrieval rate (%)	2.38% (17/713)	1.83% (10/545)	0.505

OS, ovarian stimulation; GnRH, gonadotrophin-releasing hormone; rFSH, recombinant follicle-stimulating hormone; E_2_, estradiol; P, progesterone; IVF, *in vitro* fertilization; ICSI, intracytoplasmic sperm injection; MII, metaphase II; 2PN, two pronuclei; OHSS, ovarian hyperstimulation syndrome.

### 3.3 Outcomes of fresh embryo transfer in the vaccinated and unvaccinated groups

Respectively 221 and 223 patients from the vaccinated and unvaccinated groups had cancelled fresh embryo transfer. As a result, 475 and 312 patients from the vaccinated and unvaccinated groups, respectively, underwent fresh embryo transfer. The thickness of the endometrium; the number and age of embryo transferred; and the IR, CPR, multiple pregnancy rate, early miscarriage rate, ongoing pregnancy rate, LBR, gestational age at delivery, and birth weight of infants were not significantly different between the vaccinated and unvaccinated groups (*p* > 0.05) (**Table 3**). We further calculated the power to illustrate false negative errors. For the power calculation, we set the target difference for categorical variables as 20%. The results showed that the powers for LBR, CPR, and ongoing pregnancy rate were 0.69, 0.77, and 0.71, respectively, which suggest that the false negative errors for our results were acceptable.

**Table 3:** Outcomes of fresh embryo transfer of the vaccinated and unvaccinated groups.

Variables	Vaccinated (n = 475)	Unvaccinated (n = 312)	*P* value
Thickness of endometrium (mm)	11.06 ± 2.37	10.82 ± 2.34	0.158
Number of embryo transferred	1.82 ± 0.38	1.83 ± 0.38	0.833
Age of embryo transferred (%)			0.102
Cleavage	92.63% (440/475)	95.51% (298/312)	
Blastocyst	7.37% (35/475)	4.49% (14/312)	
IR (%)	38.73% (335/865)	34.21% (195/570)	0.083
CPR (%)	55.79% (265/475)	51.28% (160/312)	0.215
Multiple pregnancy rate (%)	26.42% (70/265)	21.88% (35/160)	0.293
Early miscarriage rate (%)	10.19% (27/265)	8.13% (13/160)	0.480
Ongoing pregnancy rate (%)	48.21% (229/475)	45.51% (142/312)	0.458
LBR (%)	46.32% (220/475)	44.55% (139/312)	0.627
Gestational age at delivery (week)	37.68 ± 2.01	37.86 ± 1.92	0.386
Birth weight (g)	2820.74 ± 601.58	2889.38 ± 625.04	0.247

IR, implantation rate; CPR, clinical pregnancy rate; LBR, live birth rate.

In the vaccinated group, nine patients had ectopic pregnancies and nine patients had late miscarriages, compared with five and three patients, respectively, from the unvaccinated group. There were 282 (137 boys and 145 girls) and 171 (87 boys and 84 girls) live-born infants in the vaccinated and unvaccinated groups, respectively. No birth defects were found in the study. The mean gestational age at delivery was 38 weeks (28–41 weeks), and the mean birth weight was 2,847 g (500–4,430 g).

### 3.4 Outcomes of fresh embryo transfer in the vaccinated subgroups

In both the dose and interval subgroups, no significant differences were found in the IR, CPR, LBR, rates of early miscarriage and ongoing pregnancy, gestational age at delivery, and birth weight of infants (*p* > 0.05) (**Table 4**).

**Table 4:** Outcomes of fresh embryo transfer in vaccinated subgroups.

Variables	Dose of inoculation				Interval between the first inoculation and OS			
	Single dose (n = 74)	Double dose (n = 275)	Triple dose (n = 126)	*P* value	< 3 months (n = 65)	3 ~ 6 months (n = 123)	> 6 months (n = 287)	*P* value
IR (%)	46.67% (63/135)	37.15% (185/498)	37.50% (87/232)	0.119	45.45% (55/121)	34.51% (78/226)	39.00% (202/518)	0.134
CPR (%)	62.16% (46/74)	54.18% (149/275)	55.56% (70/126)	0.470	63.08% (41/65)	51.22% (63/123)	56.10% (161/287)	0.293
Early miscarriage rate (%)	6.52% (3/46)	11.41% (17/149)	10.00% (7/70)	0.631	7.32% (3/41)	6.35% (4/63)	12.42% (20/161)	0.323
Ongoing pregnancy rate (%)	56.76% (42/74)	46.18% (127/275)	47.62% (60/126)	0.268	56.92% (37/65)	45.53% (56/123)	47.39% (136/287)	0.300
LBR (%)	56.76% (42/74)	44.00% (121/275)	45.24% (57/126)	0.143	56.92% (37/65)	43.09% (53/123)	45.30% (130/287)	0.167
Gestational age at delivery (week)	37.62 ± 1.58	37.64 ± 2.17	37.81 ± 1.98	0.852	37.62 ± 1.83	37.72 ± 2.32	37.68 ± 1.94	0.976
Birth weight (g)	2755.42 ± 537.38	2851.39 ± 620.85	2810.00 ± 613.51	0.576	2750.69 ± 551.88	2882.01 ± 604.02	2817.50 ± 616.15	0.500

IR, implantation rate; CPR, clinical pregnancy rate; LBR, live birth rate.

## 4 Discussion

This study found that the inactivated COVID-19 vaccine did not affect the ovarian response to OS, the quality of oocytes and embryos, the LBR, and the birth weight of infants, and neither did the dose and interval of vaccination. Therefore, the vaccine appears to have no effect on the outcomes of IVF-ET.

Ovarian response is an important factor affecting the success rate of IVF-ET (33). In this study, the duration and dose of rFSH, the number of oocytes, the rates of blastocyst and high-quality blastocyst, and the numbers and rates of MII, 2PN, cleavage embryos, and high-quality cleavage embryos were similar for the vaccinated and unvaccinated groups. Another study also found no significant differences in the duration and dose of gonadotrophin between women with or without inoculation with the inactivated COVID-19 vaccine (214 *vs.* 340) (23). Dong et al. found no significant differences in the number of oocytes retrieved, the rates of fertilization, and the cleavage embryo, high-quality embryo, and blastocyst among four groups (both partners vaccinated with the COVID-19 vaccine or not, and only women or men vaccinated) (23). Other studies also reported similar numbers of oocytes, MII oocytes, 2PN, and cleaved embryos and high-quality embryos, as well as blastocyst rates, between vaccinated and unvaccinated groups (24, 25). A recent study has even shown that more oocytes were retrieved following inoculation of the COVID-19 mRNA vaccine, although the number of MII oocytes remained similar (34). Therefore, we propose that the inactivated COVID-19 vaccine may not significantly impact the ovarian response and the quality of oocytes and embryos in IVF-ET.

LBR is an important outcome of IVF-ET. Endometrial receptivity is a key factor that affects the LBR in IVF-ET (35). Although SARS-CoV-2 has not yet been isolated from human endometrium, the endometrium may still be susceptible to SARS-CoV-2 infection, particularly during the period of implantation (36, 37). The expression of the *ACE2* and *TMPRSS4* genes (both associated with viral infection) in the human endometrium may facilitate SARS-CoV-2 infection. During embryo implantation, their increased expression from the proliferative phase to the secretory phase may confer an increased risk for SARS-CoV-2 infection (36). Therefore, vaccination may be beneficial to reducing the risk of SARS-CoV-2 infection of the endometrium. However, it remains unclear whether the inactivated COVID-19 vaccine could affect the receptivity of the human endometrium. Studies have reported that women vaccinated with the inactivated COVID-19 vaccine had comparable LBR, rates of ongoing pregnancy, and clinical pregnancy compared with those unvaccinated in FET (27, 28). For the IVF/ICSI-ET treatment cycle, no influence on LBR was found (29). We also found that LBR was not affected by the inactivated COVID-19 vaccine, which suggests no significant risk of the inactivated COVID-19 vaccine to endometrial receptivity and pregnancy. Nevertheless, these results should be interpreted with caution considering the moderate power value of LBR (0.69); moreover, a larger sample size is required for further study.

There has been no consensus over the optimal interval between the vaccination and IVF-ET. The European Society of Human Reproduction and Embryology has recommended postponing the ART treatment for up to 2 months after the vaccination (38). The American Society for Reproductive Medicine has suggested that, given the time for recovery from the common side effects of the vaccine, vaccination should be avoided at least 3 days prior and after the oocyte retrieval and embryo transfer (39). Experts from China have recommended couples with stable immune response to undergo ART treatment 1 month after vaccination (40). We found similar CPRs for the three interval groups with IVF treatment (<3, 3–6, and >6 months), which is in keeping with a previous report (23). Therefore, we propose that IVF-ET should not be postponed due to the vaccination.

A study in China found that neither a single nor a double dose of an inactivated COVID-19 vaccine impacted the LBR and the birth height and weight of newborns in FET, but did not report on the triple dose as most patients were not yet inoculated with the third vaccine (27). We also found no significant differences in the IR, CPR, early miscarriage rate, ongoing pregnancy rate, LBR, gestational age at delivery, and birth weight of infants with the single, double, and triple doses of inoculations. We propose that the administration of a booster dose of an inactivated COVID-19 vaccine may be safe before IVF-ET. However, further studies are warranted to confirm these results and to explore their long-term effects.

This study compared the effects of vaccination with different doses and intervals on the LBR, which has not been reported previously. It has provided evidence of the safety of inactivated COVID-19 vaccines and could contribute to the improvement of existing studies. The limitations of this study included the small sample size for the single-dose group and the interval groups (<3 months) and that all patients were from a single center. Therefore, our findings should be confirmed in multicenter studies with a larger sample size. Furthermore, this study was conducted only on Chinese women, and the results may not be directly extrapolated for other ethnic/racial populations and/or vaccine types. More studies should be conducted on the follow-ups and collect additional data (cumulative pregnancy outcomes) for a more comprehensive analysis.

## 5 Conclusion

In summary, this study found that inoculation with the inactivated COVID-19 vaccine before IVF-ET did not affect the ovarian response, the quality of oocytes and embryos, the LBR, and the birth weight of infants, and neither did the dose and interval of vaccination. Therefore, the inactivated COVID-19 vaccine is safe for patients undergoing IVF-ET and should be inoculated before IVF-ET.

## Data Availability

The data that support the findings of this study are available on request from the corresponding author upon reasonable request. Requests to access these datasets should be directed to YG, gongyan0619@163.com.
